# Epigenetically inactivated *RASSF1A* as a tumor biomarker

**DOI:** 10.17305/bjbms.2020.5219

**Published:** 2021-08

**Authors:** Dora Raos, Monika Ulamec, Ana Katusic Bojanac, Floriana Bulic-Jakus, Davor Jezek, Nino Sincic

**Affiliations:** 1 Department of Medical Biology, University of Zagreb School of Medicine, Zagreb, Croatia; 2 Scientific Group for Research on Epigenetic Biomarkers, University of Zagreb School of Medicine, Zagreb, Croatia; 3 Scientific Centre of Excellence for Reproductive and Regenerative Medicine, University of Zagreb School of Medicine, Zagreb, Croatia; 4 Ljudevit Jurak Clinical Department of Pathology and Cytology, Sestre Milosrdnice University Hospital Center, Zagreb, Croatia; 5 Department of Pathology, University of Zagreb School of Dental Medicine and School of Medicine, Zagreb, Croatia; 6 Department of Histology and Embryology, University of Zagreb School of Medicine, Zagreb, Croatia

**Keywords:** *RASSF1A*, epigenetic alteration, DNA methylation, biomarker

## Abstract

*RASSF1A*, one of the eight isoforms of the *RASSF1* gene, is a tumor suppressor gene that influences tumor initiation and development. In cancer, *RASSF1A* is frequently inactivated by mutations, loss of heterozygosity, and, most commonly, by promoter hypermethylation. Epigenetic inactivation of *RASSF1A* was detected in various cancer types and led to significant interest; current research on *RASSF1A* promoter methylation focuses on its roles as an epigenetic tumor biomarker. Typically, researchers analyzed genomic DNA (gDNA) to measure the amount of *RASSF1A* promoter methylation. Cell-free DNA (cfDNA) from liquid biopsies is a recent development showing promise as an early cancer diagnostic tool using biomarkers, such as *RASSF1A*. This review discusses the evidence on aberrantly methylated *RASSF1A* in gDNA and cfDNA from different cancer types and its utility for early cancer diagnosis, prognosis, and surveillance. We compared methylation frequencies of *RASSF1A* in gDNA and cfDNA in various cancer types. The weaknesses and strengths of these analyses are discussed. In conclusion, although the importance of *RASSSF1A* methylation to cancer has been established and is included in several diagnostic panels, its diagnostic utility is still experimental.

## INTRODUCTION

Cancer is a significant cause of mortality worldwide, despite the tremendous progress that has been made in treatments [1]. One constant, however, is that for the successful treatment of cancer, early diagnosis is crucial. Therefore, molecular biomarkers, which can be sensitive enough to detect initial malignant transformations and progressions, are extensively studied. Biomarker candidates are developed from the knowledge of cancer development mechanisms, which involves both genetic and epigenetic abnormalities. Epigenetic abnormalities may allow early tumor detection by noninvasive methods, which would be of significant importance.

In tumor diagnosis and screening, tissue biopsies are still standard in the procedure. They can be invasive, challenging to perform, and generally are not suitable for screening. In contrast, liquid biopsies, or sampling and analysis of non-solid biological tissue such as blood, represents a less invasive diagnostic procedure with the potential to facilitate the early detection of the tumor [2,3]. Therefore, liquid biopsies as cancer biomarker sources are a quickly developing research topic. Most studies regarding liquid biopsies focus on investigating circulating tumor cells and cell-free DNA (cfDNA) released into the bloodstream. CfDNA represents small fragments of DNA released from the normal or tumor tissue as a consequence of cellular apoptosis or necrosis [4,5]. The cancer patients usually have higher cfDNA concentrations in their bloodstream because of increased tumor volume associated with increased cell apoptosis and necrosis [6]. Although blood (plasma and serum) is the most investigated body fluid, the search for specific tumor biomarkers includes other body fluids as well, including urine and saliva [7].

DNA methylation is traditionally the most investigated epigenetic modification [8], and it can be experimentally identified in liquid biopsies. Abnormal DNA methylation may be classified as hyper- or hypomethylation. For the last two decades, DNA methylation patterns of tumor suppressor genes have been investigated as potential biomarkers for many types of cancer. In that time, research has found that global DNA hypomethylation and regional hypermethylation, frequently affecting the promoter region of tumor suppressor genes, are characteristic in cancer [9,10]. One of the highlighted and most-researched biomarkers in liquid biopsies is *RASSF1A*.

*RASSF1A* gene is within the region of the 3p21.3 chromosome, which is sensitive to genetic and epigenetic changes in many tumors. Loss of heterozygosity (LOH) often occurs in this region, and promoter hypermethylation represents the mechanisms that lead to loss of *RASSF1A* gene expression. It is important to note that *RASSF1A* is more frequently inactivated by promoter hypermethylation than by LOH [11]. Aberrant *RASSF1A* methylation was detected in various cancers, including breast cancer, lung cancer, gastrointestinal cancer, prostate cancer, and testicular germ cell tumors. In some cancers, *RASSF1A* is still expressed but in suboptimal or supra-optimal concentration, which leads to disturbed signal transduction and consequent malignant transformation [12].

**FIGURE 1:**
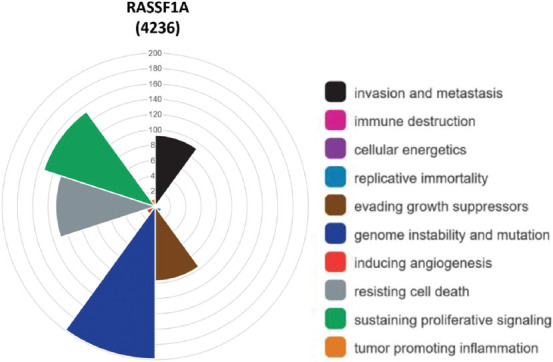
Distribution of hallmarks of cancer for *RASSF1A*. In 4236 scientific articles was detected that *RASSF1A* expression contributes the most to genome instability and mutation, and sustains proliferative signaling. Furthermore, it enables the cell to resist death, to evade growth suppressors and favors invasion of cancer and metastasis

The Ras association domain family 1 isoform (*RASSF1A*) is a tumor suppressor gene that belongs to the C-terminal RASSF family. *RASSF1* gene has eight isoforms, of which *RASSF1A* and *RASSF1C* are the most abundantly expressed [12]. These two isoforms are omnipresent in the normal cells, where they localize microtubules and regulate cell growth. *RASSF1A* is activated by mitogenic stimuli and KRAS seems to be main RASSF1A activator upon mitogenic stimulation [12]. *RASSF1A* responds to above described stimuli by regulating the cell cycle progression, apoptosis, and microtubule stability, while *RASSF1C* is involved in DNA damage response. Normally, *RASSF1C* is anchored to the the DAXX, a Death domain-associated protein and localized in the nuclei. DNA damage leads to DAXX degradation and *RASSF1C* releases into the cytoplasm where it activates the c-Jun N-terminal kinases (JNK) pathway [14]. However, in tumorigenesis. *RASSF1C* acts as an oncogene, which directly contrasts to *RASSF1A*, a tumor suppressor (Figure 1) [13].

### Epigenetic inactivation of *RASSF1A*

**FIGURE 2:**
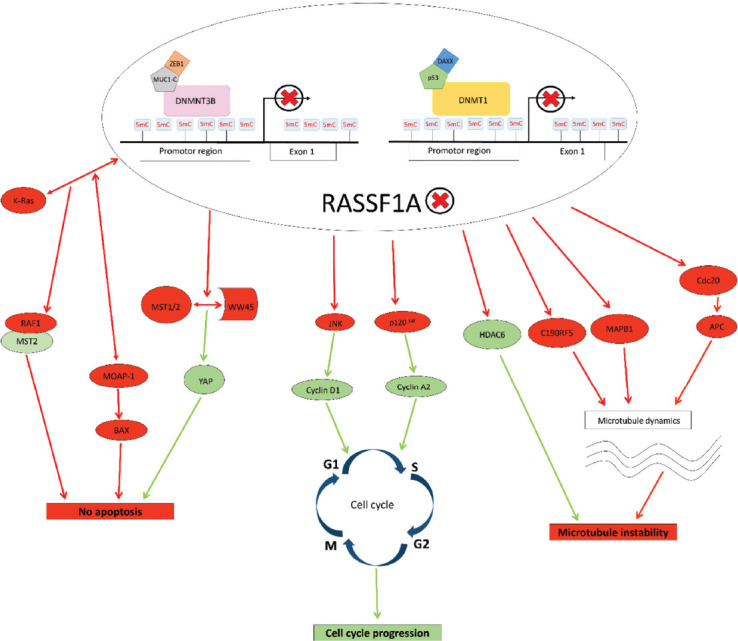
Impact of epigenetic inactivation of *RASSF1A* to cell signaling pathways. Red arrows represent disturbed or deactivated, while green ones represent activated signaling pathways. When expressed normally, *RASSF1A* causes repression of cyclin A2 and cyclin D1, which results in cell cycle arrest. *RASSF1A* also modulates apoptosis. Interactions of *RASSF1A* with K-Ras activates the MST2-LATS1 apoptotic pathway, i.e., *RASSF1A* modulates the RAF-1 activity due to competition with MST2 for RAF-1 binding. Also, the interaction of *RASSF1A* with K-Ras, enhances the interaction of *RASSF1A* and MOAP-1, promoting *RASSF1A*’s ability to induce BAX translocation to the mitochondria and cell death. *RASSF1A* binds to MST1/2 with adaptor protein WW45 that causes phosphorylation, respectively, leading to YAP phosphorylation and inhibition. Additionally, *RASSF1A* plays an important role in microtubule stability, by inhibiting HDAC6 (histone deacetylase 6), which results in an increase of acetylated microtubules, that are more stable. *RASSF1A* binds to microtubule-associated proteins (MAPs) which regulate microtubule stability. If *RASSF1A* is epigenetically inactivated, it causes microtubule instability, repression of apoptosis, and progression of the cell cycle, which favors tumorigenesis

The *RASSF1* locus is modulated by two CpG islands, A and C; CpG island A is in the regulatory region of *RASSF1A*. The methylation of CpG islands A has been detected in normal tissue and does not affect gene expression. On the other hand, hypermethylation was associated with loss of *RASSF1A* expression [13]. DNA methyltransferases (DMNTs) mediate the methylation of these CpG islands. When DMNTs are dysregulated, its actions in cancer cells lead to the epigenetic silencing of *RASSF1A*. It has been shown that MUC1-C or p53 protein bind to the *RASSF1A* promoter and consequently activates their corepressors ZEB1 and DAXX. MUC1-C-ZEB1 complex recruits DMNT3B, while p53-DAXX complex recruits DMNT1. This activity causes CpG island’s methylation in the *RASSF1A* promoter region and the subsequent loss of *RASSF1A* expression [16]. In cancer cells, loss of *RASSF1A* because of this cascade leads to the binding of *RASSF1C* to the *RASSF1A* effectors, which, in turn, favors tumorigenesis [14,15]. Epigenetic inactivation of *RASSF1A* causes disturbance in various signaling pathways, as outlined in Figure 2.

This review summarizes the current knowledge on DNA methylation of *RASSF1A* in genomic DNA (gDNA) and cfDNA in various tumors. We provide a comprehensive assessment of the diagnostic and prognostic value of *RASSF1A* in cfDNA and gDNA, highlighting some potential benefits. We will also be discussing some limitations regarding the utility of *RASSF1A* methylation as a tumor biomarker in clinical practice.

### Breast cancer

Breast cancer (BC) is the most frequent malignancy among women worldwide, and early detection of BC broadly defines disease management planning and the course of treatment. When mammography was implemented as a routine diagnostic procedure for BC, there was an overall increase in early detection. However, there were unintended consequences, along with that improvement. According to Nelson *et al*. [17], the rates of false-positive mammography results were the highest for women between 40 and 49 years old and decreased with increasing age. Also, rate of false-positive mammography results were statistically significantly higher for women with specific risk factors then for those without them [17]. Mammography and ultrasound cannot always differentiate benign and malignant lesions [18,19], so women with false-positive results received biopsies. Therefore, there is significant research interest in *RASSF1A* methylation in gDNA and cfDNA as a possible BC biomarker.

According to UALCAN, the β value of *RASSF1A* methylation in BC patients is 0.311, hypermethylated compared to healthy tissue (β value is 0.22). *RASSF1A* methylation frequency in BC tissue samples in gDNA was detected in around 65.0% of BC tissue samples [20,21]. Regarding cfDNA, the *RASSF1A* methylation frequency was detected in the range from 63.3% [20,22] to 16.6% [21,23]. All studies that detected such a wide range of *RASSF1A* methylation in cfDNA used the same method (methyl-specific PCR) for *RASSF1A* methylation analysis. The only difference was specific primers, which might explain diversity in *RASSF1A* methylation frequency and show a specific site in the promoter region, which is more methylated than the others and might have a greater impact on BC tumorigenesis. Indeed, two different primer pairs gave different results regarding the methylation frequency of *RASSF1A* in the same study [20]. Different technologies for methylation detection could give different results than reported by Skalski and Paulszczak [24].

The reported results of variability in the *RASSF1A* methylation analysis in cfDNA of BC patients show that the *RASSF1A* methylation pattern in cfDNA requires further investigation before it is considered a possible BC biomarker.

In addition to using different specific primer pairs, the potential challenge could be that the entire first *RASSF1A* exon contains a CpG island [25]. This CpG island is methylated in normal breast tissue [26], and the methylation of *RASSF1A* was also detected in the promoter region in some healthy control samples. Methylation of *RASSF1A* in healthy controls could indicate that methylated *RASSF1A* would be useless as a breast cancer biomarker because it does not differentiate enough between healthy tissue and tumor tissue. However, *RASSF1A* methylation was not as frequent as seen in BC patient samples. Based on these results, the authors pointed out that *RASSF1A* methylation in BC occurs progressively from the first exon to the promoter region and is beginning early in breast tumorigenesis [26]. This finding can help us understand why some studies detected hypermethylated *RASSF1A* in healthy controls and consequently excluded *RASSF1A* from further investigation [27].

Strikingly, *RASSF1A* was more frequently methylated in the serum of healthy controls than in the serum of patients with benign breast disease [22,28], a particularly important finding when considering using these patients for controls. This finding may lead to incorrect associations regarding BC patients’ methylation patterns and healthy, tumor-free women.

### Clinicopathological parameters

Hypermethylated *RASSF1A* was found associated with clinicopathological parameters, meaning that besides diagnostic value, it could have prognostic value as well. A critical clinicopathological parameter for BC diagnosis, treatment, and prognosis is the estrogen receptor status (ER) since ER-positive (ER+) BC tumors are less aggressive. Methylation of *RASSF1A* in gDNA and cfDNA is associated with ER and progesterone receptor status (PR). Hypermethylated *RASSF1A* was detected with high frequency in ER-positive/PR-positive (ER+/PR+) (20,29,30). Furthermore, higher methylation levels of *RASSF1A* were detected in ER+/PR+ BC tissue samples when compared to ER+/PR- tumors. [31]. Hypermethylated *RASSF1A* from gDNA and cfDNA was strongly associated with tumor size and poor prognosis. However, the literature is not consistent as some authors did not detect any significant association between methylation in cfDNA or gDNA with clinicopathological parameters [21]. Furthermore, DNA methylation of *RASSF1A* seemed to be associated with lymph node metastasis (LN) [32], showing a similar methylation frequency in both gDNA and cfDNA [20].

### Future clinical utility

*RASSF1A* methylation status as cancer biomarker has high sensitivity and specificity for detecting BC in gDNA, while in cfDNA, *RASSF1A* biomarker specificity was 100.0%, but sensitivity was low 78.83% [22,31]. However, no single gene was hypermethylated in all BC patients, and a specific gene panel should be favored in the diagnosis [33]. Panel testing for aberrantly methylated genes in BC, which included *RASSF1A* in gDNA and cfDNA, resulting in a better diagnostic performance compared to *RASSF1A* methylation analysis exclusively [31]. Furthermore, when analyzing a panel of genes, the panel had a higher diagnostic-sensitivity than mammography for tumors ≤1 cm. For tumors >2 cm, the sensitivity was lower than for mammography [28]. These findings suggest a gene panel has the potential for use in early diagnosis protocols. The same panel could discriminate between BC patients and healthy women with 79.6% sensitivity and a specificity of 72.4%. Importantly, methylation panel testing was able to distinguish BC patients from women with benign breast diseases, with high specificity (78.1%) and even higher sensitivity (82.4%) (28).

### Lung cancer

According to GLOBOCAN 2018, lung cancer (LC) has the highest mortality rate of all cancers worldwide. Because of the absence of symptoms in the early stages of the disease, approximately 70% of patients are diagnosed in an advanced stage. Low-dose computed tomography (LD-CT) is currently considered the best LC screening method available [34]. However, LD-CT screening of high-risk smokers had shown 24% positivity among which 96% of the individuals tested positive were false positive [35]. Therefore, there is a need for a new, more accurate screening approach for LC, which can provide early detection.

As with BC, aberrant methylation of *RASSF1A* was detected in LC. Compared to BC, hypermethylated *RASSF1A* was detected only in patients with LC, compared to none in healthy control samples [36–39]. This evidence suggests *RASSF1A* is more discriminative between LC and healthy controls than between BC and healthy controls. Significantly, different *RASSF1A* methylation frequencies were observed between small-cell (SCLC) and non-small cell lung cancer (NSLC) [40]. *RASSF1A* methylation frequency in gDNA and cfDNA was higher among SCLC patients than among NSCLC [41,42]. As SCLC represents a more aggressive LC than NSLC, higher *RASSF1A* methylation frequency could be linked to more aggressive LC [40].

Furthermore, studies with LC patients for cfDNA testing potential detected that *RASSF1A* methylation in gDNA was not always accompanied by methylation in cfDNA [36]. This relationship should be considered, since methylated *RASSF1A* in cfDNA could be detectable only in already advanced stages of the disease.

### Clinicopathological parameters

Smoking represents a high risk of developing LC, and research has shown that the methylation of *RASSF1A* was higher in smokers than in non-smokers [43]. Even the duration of the number of years smoking was associated with hypermethylation of *RASSF1A*, detected with an observational study [39]. Interestingly, LC from men exhibited higher methylation levels of *RASSF1A* than those from women (7.5% *versus* 17.9%, P < 0.01) [44]. This finding indicates that *RASSF1A* methylation could have different efficacies for men than women.

Methylation of *RASSF1A* was determinative between NSCLC than SCLC [45,46]. This difference was also detected by UALCAN, where β value of adenocarcinomas, one of the main subtypes of NSCLC was slightly higher than of SCLC, i.e., mean of β is 0.265 in adenocarcinomas and 0.243 in SCLC. As adenocarcinoma is the most common type of LC [47], *RASSF1A* methylation could be used to diagnose most LC, particularly among non-smokers low-risk populations [48]. Considering overall survival, patients with hypermethylated *RASSF1A* have lower overall survival rates than patients without hypermet0hylated *RASSF1A* [43,49].

There is also a significant association was detected between *RASSF1A* and TNM stages. When TNMs were investigated separately, there was no significant difference. However, when TNM I and II were observed as one group vs. TNM III, methylation of *RASSF1A* was significantly associated with TNM III [40,43]. *RASSF1A* levels in cfDNA from blood plasma were significantly higher in LC patients with distant metastatic disease [50]. Based on the presented studies, *RASSF1A* seems like a promising prognostic biomarker for LC. However, the research is inconsistent for associations between clinicopathological parameters and *RASSF1A* methylation in tissue or blood samples (37,44,45,51). This discrepancy was often explained that the number of samples was significantly lower than in studies in which *RASSF1A* hypermethylation was associated with the clinicopathological parameters.

### Future clinical utility

Specific genes’ hypermethylation can be detected with other noninvasive sampling techniques, including plasma and sputum [52], which is very useful for LC. Sputum contains exfoliated epithelial cells where hypermethylation of *RASSF1A* was detected. *RASSF1A* methylation individually showed high specificity (96.5%), while the sensitivity was significantly lower (42.5%) [53]. Regarding cfDNA, *RASSF1A*, together with *p16INK4a*, represents the most frequently reported gene in blood-based liquid biopsies displaying 22%–66% sensitivity and 57%–100% specificity for LC detection [54]. However, when incorporated into the methylation gene panel, both the sensitivity and specificity for *RASSF1A*, together with *p16INK4a*, were significantly higher in cfDNA [50,55]. Besides, the sensitivity of hypermethylated *RASSF1A* in gDNA was higher when *RASSF1A* was incorporated into a methylation gene panel, while specificity was more or less the same [53,56]. Bronchial lavage fluid (BLAF) represents a less invasive method than tissue biopsy and is an excellent candidate for a source of biomarkers for early detection. In gDNA from BLAF and tumor tissue sample, similarly to gDNA from sputum, the methylation gene panel’s sensitivity increased significantly [57,58]. Methylation panel of *SHOX2* and *RASSF1A* in BLAF demonstrated dominantly higher sensitivity than cytological examination or analysis of serum biomarker carcinoembryonic antigen, which is both used for cancer screening [59]. Although BLAF sampling represents an invasive method with potential complications, it represents a promising diagnostic tool for detecting LC’s early detection. Due to LC’s nature, where sputum and BLAF represent a better source of LC biomarkers than blood, methylation of *RASSF1A* in gDNA showed a better diagnostic value than in cfDNA.

### Gastrointestinal cancer

Like LC, gastrointestinal cancer (GC) causes high mortality worldwide since they are diagnosed at an advanced stage when the prognosis is poor, and the treatment options are limited [60]. The diagnosis of GC usually requires endoscopy, followed by a biopsy of tissue from a suspicious area. Therefore, it represents an invasive and unpleasant diagnostic method that many people avoid until the disease’s clinical manifestations. GC is a very heterogeneous and complex disease that involves many genetic and epigenetic alterations. Therefore, biomarkers useful for molecular diagnosis would enable earlier detection and diagnosis. In studies of methylation patterns of different genes in GC such as gastric, liver, and colorectal cancer, aberrant methylation of *RASSF1A* has emerged as a promising biomarker.

In colorectal cancer, *RASSF1A* was silenced by hypermethylation in aberrant crypt foci (ACF) [61]. ACF represents the earliest morphologically indeterminate mucosal abnormality in the colon, which can progress to colorectal cancer (CRC), meaning hypermethylated *RASSF1A* could be used for the detection of possible precancerous subsets [62]. Methylation of *RASSF1A* in morphologically indeterminate mucosal abnormality in the colon was confirmed in studies where gDNA from adjacent tumor-free mucosal tissue served as a control and hypermethylated *RASSF1A* was found in these controls [63]. The situation is different in gastric cancer, where hypermethylated *RASSF1A* was detected in cfDNA of patients with benign gastric disease, such as chronic gastritis, gastric ulcers, and benign polyp, non-malignant adenoma, and ulcerative colitis, but in none of the healthy tumor-free control [64]. Nevertheless, this evidence suggests hypermethylated *RASSF1A* plays a role in early gastric carcinogenesis, which may be initiated in adjacent tumor-free tissue near the tumor region. In hepatocellular carcinoma (HCC), the most common liver malignancy, hypermethylated *RASSF1A*, was also detected in cancer patients’ tissue and serum samples, but was not detected in healthy controls [65,66]. In the early stages, a diagnosis of HCC, when the treatment is still favorable, seems complicated because about 75,0% HCCs coexist with liver cirrhosis [67]. However, a comparison of serum samples of patients suffering from liver cirrhosis to those with HCC showed that *RASSF1A* hypermethylation is relatively specific to HCC [68,69]. A higher *RASSF1A* methylation frequency was found in cfDNA of patients with chronic hepatitis C infection than in healthy controls, but this increase was lower than in HCC patients [70]. Hepatitis C infection could promote HCC development through disruption of the *RASSF1A* methylation frequency/pattern.

### Clinicopathological parameters

In the context of the possible prognostic biomarker for gastric, colorectal, and liver cancer, *RASSF1A* showed potential. Hypermethylated *RASSF1A* in gDNA was associated with TNM stages in gastric cancer, where frequencies of hypermethylated *RASSF1A* in patients with stages III and IV were significantly higher than in stages I and II [71]. A significant association of *RASSF1A* promoter hypermethylation and more advanced stages of the disease was also observed in cfDNA from gastric cancer patients [64]. Hypermethylated *RASSF1A* was associated with advanced stages in liver cancer [72]. A significant association between LN metastasis and *RASSF1A* methylation was found in cfDNA of gastric cancer patients [64]. Hypermethylated *RASSF1A* was linked to poor prognosis and low overall survival in liver cancer and CRC patients [73–75], and the same correlation was found in CRC, although in gDNA [63]. On the other hand, in one study on gastric and colorectal adenocarcinoma patients, no significant difference in *RASSF1A* methylation between preoperative serum samples and four weeks postoperative serum samples was found [42] and implied it not be useful in the surveillance prediction of gastric and colorectal adenocarcinoma patients.

### Future clinical utility

An elevation in alfa-fetoprotein (AFP) level is a widely used serum marker for HCC screening. The cut-off value of serum concentration of 20 ng/mL is commonly used to differentiate HCC patients from healthy adults in clinical studies [76]. However, AFP specificity and sensitivity are limited because the AFP level is elevated in non-malignant liver diseases, like inflammation and liver cirrhosis. Hypermethylation of *RASSF1A* in cfDNA of HCC patients had showed high specificity in discrimination of HCC patients from healthy controls [77]. Moreover, individual *RASSF1A* methylation status displayed good diagnostic performance regarding discrimination between HCC patients and patients with hepatitis C [70]. Incorporation of *RASSF1A* into methylation panel with different genes further improved diagnostic performance [77,78]. *RASSF1A*, *APC*, *GSTP1*, and *SFRP1* gene panel has successfully discriminated HCC patients from healthy controls with 84,7% sensitivity and 87,8% specificity [79]. Methylation statuses of *RASSF1A*, *BVES*, and *HOXA9* in serum, when analyzed together, showed 73,5% sensitivity and 91,1% specificity for distinguishing between HCC and chronic hepatitis patients [69]. Additionally, when aberrantly methylated *APC*, *COX2*, and *RASSF1A*, were combined with other epigenetic marker miR-203, 75,0% of HCC cases were underdiagnosed by AFP measurement [80].

### Methylation of *RASSF1A* in other cancers

Besides the association of *RASSF1A* methylation with BC, LC, and GC, cancers with the highest incidence worldwide, the diagnostic and prognostic value of *RASSF1A* methylation were also investigated in other malignancies. Patients with renal cell cancer (RCC) and prostate cancer (PC) could be symptom free in the early stage, while diagnosing the disease has already progressed. Also, routine diagnostics methods for RCC and PC involve substantial patient discomfort and have variable sensitivity [81]. Given the shortcomings of current screening methods and predictive biomarkers, the development and implementation of useful biomarkers for early detection are crucial.

For bladder cancer (BCA), a similar *RASSF1A* methylation level was found in cfDNA from BCA patients and patients with non-malignant bladder disease [82]. These results question the utility of methylation of *RASSF1A* as a biomarker for BCA because of low specificity [83]. In RCC [84–86] and PC [50], a wide range of *RASSF1A* methylation frequency in cfDNA was reported. The variable *RASSF1A* methylation frequency ratio was also detected in gDNA from head and neck squamous cell carcinoma (HNSCC) [87]. A discrepancy regarding HNSCC may arise from the lower importance of *RASSF1A* hypermethylation in HNSCC tumorigenesis. Indeed, hypermethylation of other genes, such as *MGMT*, *DAPK*, and *p16*, was more frequently detected in HNSCC patients [88]. Methylation of *RASSF1A* in cfDNA from HNSCC patients has not yet been reported. In testicular germ cell tumor (TGCT), methylation of *RASSF1A* in gDNA was discriminative between one of the TGCT subgroups, seminomas, and healthy tissue [89]. In contrast, *RASSF1A* methylation in cfDNA was not investigated to date.

Most cancers are presented with heterogeneous groups of various more or less similar cellular entities, so discrimination between tumor subtypes is crucial. *RASSF1A* seems to meet this challenge. Combined methylation analysis of two genes, *HOXA9/RASSF1A*, had successfully distinguished two subgroups, nonseminomas and seminomas in TGCT diagnostics [90,91]. In BCA, significantly higher methylation was detected in muscle-invasive BCA than noninvasive one [66,83]. Regarding RCC, *RASSF1A* methylation in gDNA had discriminated well clear cell renal carcinoma (ccRCC) from papillary renal carcinoma (pRCC) patients. Higher methylation frequency was detected in pRCC [92,93], but the methylation of *RASSF1A* in cfDNA was not associated with RCC’s histological subtypes [86].

Besides diagnostic value, the association of *RASSF1A* methylation and clinicopathological parameters was proven, indicating its prognostic value in several tumors. A strong association of hypermethylated *RASSF1A* in gDNA and cfDNA, respectively, and aggressive PC was detected, where hypermethylated *RASSF1A* correlated with Gleason score and serum PSA [94,95]. Hypermethylation of *RASSF1A* was linked to high grade and advanced-stage tumors of cervical cancer [96], HNSCC [97], RCC [98], BCA (66,81,99), melanoma [100], and brain tumor [101]. Hypermethylated *RASSF1A* in TGCT was associated with chemotherapy resistance [102] and lower response to treatment [103]. In tumors of the central nervous system (CNS), hypermethylated *RASSF1A* in cfDNA was found more frequently in glial tumors than in metastatic CNS neoplasms [104]. Hypermethylated *RASSF1A* was also linked to a lower overall survival rate in HNSCC [71].

Hypermethylated *RASSF1A* was associated with risk factors for several tumors. Tobacco use and human papillomavirus (HPV) infection are some primary risks for HNSCC [105,106], and it was shown that methylation of *RASSF1A* can successfully distinguish smokers and non-smokers [107]. HPV positive patients had presented significantly higher methylation of *RASSF1A* than HPV negative patients [108].

In the investigation of hypermethylated *RASSF1A* in several cancers, adjacent non-malignant tissue, used as a control group, could present a challenge. As malignant transformation is already present in these patients’ organisms, it could reflect the epigenome in the more or less healthy region near the tumor [109]. The possible reflection of malignant transformation on epigenome in the adjacent non-malignant tissue could explain why several studies had found high *RASSF1A* methylation frequency in tumor tissue and adjacent non-malignant tissue, e.g., in RCC (85,110,111) and HNSCC [112–114]. The reported level of *RASSF1A* DNA methylation should be contextualized with lifestyle as well. A lot of HNSCC patients have been smoking, and it was shown that DNA methylation of *DAPK1*, *p16INK4a*, and *RASSF1A* is significantly different in smokers and non-smokers [107], regardless of the presence of malignant tissue. Therefore, DNA methylation patterns of genes in adjacent non-malignant tissue could be altered because of smoking, but not only because of cancer. Adjacent non-malignant tissue does not represent an adequate control group for DNA methylation studies [115].

### *RASSF1A* DNA methylation in gDNA vs. cfDNA

DNA methylation is nowadays widely used to detect cancer, triage, screening, or surveillance. Methylation patterns can be detected in gDNA or cfDNA. Since gDNA can be isolated from circulating tumor cells, its analysis can be used in liquid biopsies samples and cfDNA. Indeed, diagnostic tests detect aberrant methylation in cancer gDNA from circulating tumor cells in liquid biopsies [116]. In the case of *RASSF1A* methylation, it has been implemented into the diagnostic test for PC detection. Namely, the ConfirmMDx test detects *GSTP1*, *RASSF1A*, and *APC* genes that exhibit increased methylation in prostate tumor tissue [117]. As tumors are very heterogeneous, simple tissue biopsy provides only a fragment of cancer cell subpopulation in the tumor, giving a misleading image of the tumor’s true cellular content. In cfDNA, all tumor cell types contribute to the total cfDNA compartment, providing more accurate tumor composition information. The potential challenge regarding cfDNA could represent rare tumor sub-populations and early stage tumors, in which cfDNA concentration could be too low, making it undetectable by a diagnostic test. Despite all these challenges, various tests for detecting aberrantly methylated genes in cfDNA were developed and integrated into clinical practice. Epi proColon detects methylated *SEPT9* in CRC patients, while COLVERA detects ­methylated *BCAT1* and *IKZF1* genes, which are associated with colorectal tumor growth. Furthermore, HCCBloodTest detects methylated *SEPT9* in plasma samples of cirrhotic patients, which are at high risk for developing HCC. The therascreen MGMT Pyro test detects methylated *MGMT*, which is associated with the treatment response in the case of glioblastoma, and Epi proLung test detects aberrant methylated *PTGER4* and *SHOX2* in cfDNA from blood plasma in LC [116].

### Challenges in translating *RASSF1A* DNA methylation as a useful cancer biomarker

**FIGURE 3:**
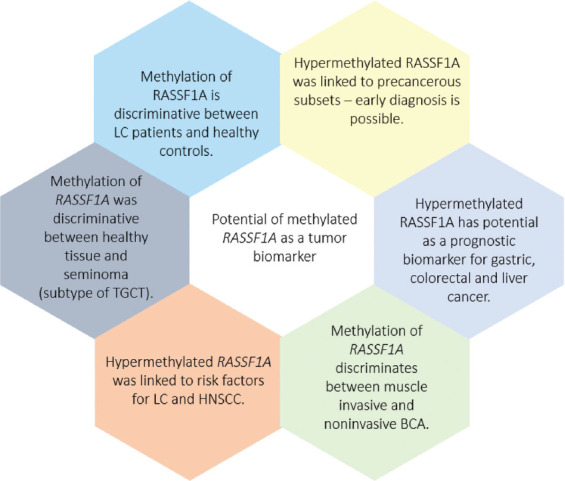
Potential of *RASSF1A* methylation as a tumor biomarker

As discussed in this review, the expression of *RASSF1A* is altered by hypermethylation in various tumors. Its methylation was extensively studied as a potential diagnostic and prognostic tumor biomarker. Indeed, based on the evidence, *RASSF1A* methylation showed an excellent diagnostic performance in distinguishing several tumors from healthy controls. It was linked to various clinicopathological parameters that pointed out its potential value in future clinical use (Figure 3).

**FIGURE 4:**
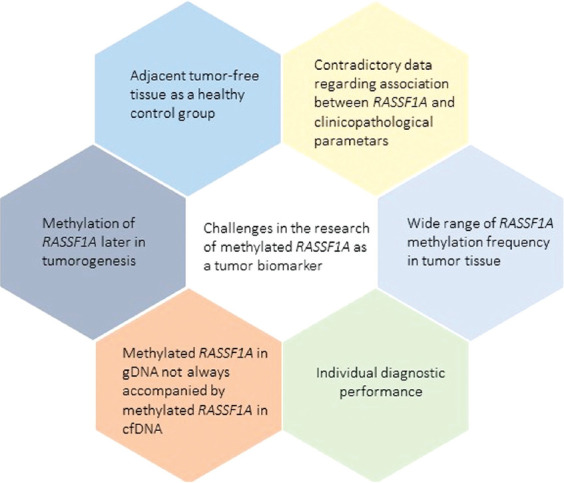
Drawbacks that presented itself in the research of methylated *RASSF1A* as a tumor biomarker. Highlighted drawbacks should be taken into account and possibly solved, before the translation of methylated *RASSF1A* as a tumor biomarker in the clinic

However, to validate the hypermethylation of *RASSF1A* as a useful cancer biomarker, some challenges should be addressed (Figure 4). A contradictory data on *RASSF1A* methylation frequency was detected in various malignant diseases for cfDNA and gDNA, which could be because different studies analyzed different CpG sites, and not all of them are equally methylated or biologically active in terms of gene expression. Regarding cfDNA, this wide range of contradictory results could be because of studies of cfDNA collected from patients with different cancer stages. The low methylation frequency could reflect early stage tumors, which are not highly vascularized and have a low necrosis rate in the tumor core.

Furthermore, there were cases where *RASSF1A* in gDNA was methylated but not in paired cfDNA samples. This could be explained by the fact that the methylation in cfDNA can be detected after the malignant cells are necrotized. In contrast, gDNA methylation can be detected when the malignant cell is still viable. Thus, the failure of methylated *RASSF1A* detection in cfDNA does not rule out the possibility of the tumor presence. It can also be that methylation of *RASSF1A* occurs later in tumorigenesis, so that cfDNA from these tumors carrying aberrantly methylated *RASSF1A* is not yet present in the blood. This may also suggest that when the methylated *RASSF1A* is detected in the peripheral blood, the tumor is present in the advanced stage. If this would be the case, detection of aberrant *RASSF1A* methylation in cfDNA might not be suitable for early diagnosis.

There are additional discrepancies between some studies where various degrees of association of *RASSF1A* methylation and clinicopathological parameters were analyzed. This inconsistency in *RASSF1A* methylation could implicate that methylation of *RASSF1A* does not play such an essential role in disease development as it was presumed. It might be that the aberrant methylation of other genes has a more significant influence on the tumorigenesis of some cancers. Furthermore, from studies of *RASSF1A* methylation in tumor tissue samples, it must be determined which tissue samples can be used as a control group. The usage of tumor-free benign disease tissue or adjacent tumor-free tissue might lead to wrong conclusions about *RASSF1A* methylation alterations in tumorigenesis. This suggests that referent healthy tissue should be used more frequently to analyze altered gene methylation in cancers.

Methylation of *RASSF1A* showed good diagnostic value individually, but in most studies, its sensitivity was low. Therefore, it was considered advantageous to use a panel of genes for cancer screening procedures since it usually provides more accurate diagnostic and prognostic information. Indeed, a gene panel of *GASP1/APC/RASSF1A* is currently used to diagnose PC. Furthermore, as aberrantly methylated *RASSF1A* was detected in various cancer types, a panel of aberrantly methylated genes that includes *RASSF1A* could be more specific for screening various cancer types.

## CONCLUSION

A body of evidence shows that epigenetic inactivation of *RASSF1A* is strongly associated with tumorigenesis and cancer behavior. However, its specificity and sensitivity are increasing when combined with other aberrantly methylated genes. *RASSF1A* DNA methylation can be a cancer biomarker, although some critical issues must be addressed before translation into routine clinical practice.
